# The Good Surgeon

**Published:** 1918-08

**Authors:** 


### THE GOOD SURGEON : THE MOST IMPORTANT FACTOR IN THE TREATMENT OF WAR WOUNDS

The surgeons and pathologists, who for four years have intensively studied war wounds, have formulated many views — many apparently contradictory views — such as, various chemical agents against no chemical agent; moist dressing against dry; heat against cold; frequent dressings against infrequent, and against both, no dressings; sunlight and electric light against occlusion; immersion versus hot air; bacteriologic control against clinical judgment; vaccines, toxins, and foreign proteins against normal reaction; wound inoculation of harmless organisms against wound sterilization; isotonic against hypertonic solutions; paste competing with paste — *bip* against *ip*, *sap* against both, and *chromatic* pastes against all. Does not the intensive study of war wounds for a short period equal and recapitulate the more leisurely study during the thirty years since Lister brought out the carbolic spray? And is there not slowly emerging from the present conflict of views the one and identical agent of successful surgery that emerged from the post-Listerian period — *the good surgeon* ?

In civil surgery in America what was the agency by which mastery was achieved over appendicitis, cholecystitis, tubal infection, adenitis? What agent succeeded best in resection of the intestine; in gastroenterostomy; in suppurating stone in the kidney; in resection of the stomach; in infection of the subcutaneous tissue? What agencies achieved survival? But *one* — the sound surgeon, who always creates opportunity. Is it possible that in these four intense years of war surgery, in which there has been accumulated more experience in traumatic surgery than during the past 30 years, we have traveled around the same circle as in civil surgery and have again found the same *surgeon* ?

By sound surgery we mean the assumption of complete inclusive responsibility for every item that enters into the result; the consideration of the wound as well as the patient; the development of an ability to read the wound as well as the man aright. It means quick, innocuous, timely intervention; it means seeing clearly the to-morrow of the wound; it means no intervention unless there is to be a net gain; it means a sharp knife, a good anesthetic, a painless innocuous dessing; it means as much respect for the tissues of the anesthetized man as for the unanesthetized man: it means a training in judgment that unerringly tells when to cut, how far to cut, when to quit cutting. It plays all the defenses and reparative forces of the patient. Good surgery is the exponent of no single method. It recognizes the anatomical and environmental situation in which chemical and physical agencies are useful. Good surgery exploits physiologic rest and fluids and sleep; gives little pain. Good surgery evokes confidence; and confidence begets rest; and rest begets restoration. Good surgery, then, makes use of antiseptics and physical forces, just as it uses incisions, counter-drainage, revisions, skin-grafting, blood-transfusion. Good surgery does not substitute an easy formula for its principles; above all, it always is dissatisfied with its work and is open to suggestion.

What can the good surgeon accomplish in war with wounds, with good opportunity but no antiseptics? Without antiseptics he can close by primary union a higher percentage of contaminated wounds than he can with antiseptics; he is able to remove damaged tissue with such accuracy that the natural defenses of the revised wound become its best antiseptic; he closes penetrated knee joints more securely without than with antiseptics; he closes penetrated skulls without, better than with, antiseptics; he operates on perforated intestines more successfully without than with antiseptics; he clears up foul and infected superficial wounds as well without as with antiseptics; he meets gas gangrene with the timely use of the knife as well without as with chemical agents. He closes healthy superficial wounds with early suture tied lightly; healthy wounds that cannot be closed by suture he closes by skin grafting, both as a healing and as a bacteriocidal policy; he closes fecal and urinary fistulae without antiseptics.

On the other hand, he realizes equally that in compound fractures with or without bone infection, in deep, recessed wounds, in pyocyaneus infection, in many other wounds, antiseptics may have great advantage, and he uses them and uses them well. In certain phases of a wound, he would use Carrel-Dakin; in another, acetic acid; in another, hot pack; in another, incision — he makes physiologic incisions today to avoid the tissue tension of tomorrow; — in another, a transfusion; in another, sunlight or electric light; in another, continuous alcohol to make a scar covering.

In the rush of a great battle, he would incise for drainage, and in addition he would make "physiologic incisions" to avoid tension that is sure to follow the next day from the inevitable infection.

But in quiet times, he would dissect out with macroscopic exactness every atom of devitalized tissue. He reads accurately not only the wound, but the patient; not only the patient, but the military situation; not only the military situation, but the condition of the infecting soil, the state of transport, his surgical assistance, and the type of nursing care — that is, he weighs accurately his chances for success. Therefore, the army medical service and the wounded man pin their hope and their faith first, last, and always to the one agency of wound treatment that in civilian surgery emerged clearly from the confusion of the Listerian period; is emerging clearly from the confusion of the four years of military surgery — *the sane, sound surgeon.*

What our army needs beyond all else is an adequate number of sound surgeons; and these men should be given every opportunity for their development. The next most important factor is — not chemical agents, but a transport so organized that the sound surgeon comes in contact with his patient at the earliest moment; and that there is provided for *each wounded man requiring operation at the front* for an average period of half an hour, a table and a good surgeon.

